# Genetic Variants of *ISL1* Gene Promoter Identified from Congenital Tetralogy of Fallot Patients Alter Cellular Function Forming Disease Basis

**DOI:** 10.3390/biom13020358

**Published:** 2023-02-13

**Authors:** Xiu-Yun Yin, Huan-Xin Chen, Zhuo Chen, Qin Yang, Jun Han, Guo-Wei He

**Affiliations:** 1 The Institute of Cardiovascular Diseases & Department of Cardiovascular Surgery, TEDA International Cardiovascular Hospital, Tianjin University & Chinese Academy of Medical Sciences, Tianjin 300457, China; 2 School of Pharmacy, Drug Research & Development Center, Wannan Medical College, Wuhu 241002, China

**Keywords:** tetralogy of Fallot, *ISL1*, genetic variant, congenital heart disease

## Abstract

Tetralogy of Fallot (TOF) is the most common cyanotic congenital heart disease in newborns. *ISL1* is a master transcription factor in second heart field development, whereas the roles of *ISL1* gene promoter variants in TOF patients have not been genetically investigated. Total DNA extraction from 601 human subjects, including 308 TOF patients and 293 healthy controls, and Sanger sequencing were performed. Four variants (including one novel heterozygous variant) within the *ISL1* gene promoter were only found in TOF patients. Functional analysis of DNA sequence variants was performed by using the dual-luciferase reporter assay and demonstrated that three of the four variants significantly decreased the transcriptional activity of *ISL1* gene promoter in HL-1 cells (*p* < 0.05). Further, the online JASPAR database and electrophoretic mobility shift assay showed that the three variants affected the binding of transcription factors and altered *ISL1* expression levels. In conclusion, the current study for the first time demonstrated that the variants identified from the *ISL1* gene promoter region are likely involved in the development of TOF by affecting the transcriptional activity and altering the *ISL1* expression level. Therefore, these findings may provide new insights into the molecular etiology and potential therapeutic strategy of TOF.

## 1. Introduction

Tetralogy of Fallot (TOF) is the most common form of cyanotic congenital heart disease in newborns, with a prevalence of 4 per 10,000 live births, accounting for up to 7–10% of congenital heart diseases (CHDs) [1,2]. TOF is characterized as a combination of abnormalities (ventricular septal defect, obstruction of the right ventricular outflow tract, override of the ventricular septum by the aortic root, and right ventricular hypertrophy) that together lead to tissue hypoxia. Although surgical correction during infancy repaired most cases of TOF and increased the survival of TOF patients [3], there is significant morbidity in adulthood, particularly with pulmonary regurgitation and arrhythmias [4].

The genetic factor has been demonstrated to be the major etiology of CHDs [5]. Accumulating evidence has indicated that the cause of TOF is complex and genetic factors are major contributors to the incidence of TOF [6]. The presence of newer genomic technologies, especially genome-wide sequencing, has identified novel TOF-specific copy number variants and revealed the genetic basis of TOF [7]. Studies have identified susceptible genes related to the etiology of TOF, such as NOTCH1 [6], FLT4 [6], *ISL1*, TBX1 [8], PIPN11 [9], and NRP1 [10]. However, the roles of these associated genes and pathways in the formation of TOF remain largely unknown.

The transcription factor *ISL1* (OMIM: 600366), which is located on chromosome 5q11.1, has been considered an important regulator of heart development. As a crucial member of the LIM homeobox family of transcriptions, *ISL1* is a marker for cardiac progenitor cells, primarily in marking undifferentiated, proliferating progenitors in the second heart field, and is essential for the proliferation, migration, and survival of cardiac progenitor cells [11,12,13]. In mice, homozygous null for *ISL1* appears to lead to serious heart development defects, including absence of right ventricle, much of the right atrium, and outflow tract [11]. Furthermore, it has been found that several mutations of *ISL1* were associated with diverse types of CHDs in humans [14,15,16,17]. It is well known that the promoter, located at 5′upstream of gene, plays a key role in gene transcriptional regulation. Accumulating evidence has reported that the variants of gene promoter region may affect the expression level of genes, leading to the development of disease [14,18,19]. However, there are no studies focusing on the variant of *ISL1* gene promoter region in TOF patients. Therefore, we hypothesized the genetic variants in *ISL1* gene promoter may contribute to the development of TOF by altering the *ISL1* gene expression level and affecting the binding ability of transcription factor. To confirm this hypothesis, we designed this study and identified variants within the *ISL1* gene promoter in patients with TOF in comparison with healthy controls and then functionally analyzed the effect of the variants on the *ISL1* gene expression.

## 2. Materials and Methods

### 2.1. Study Population

**Figure 1:**
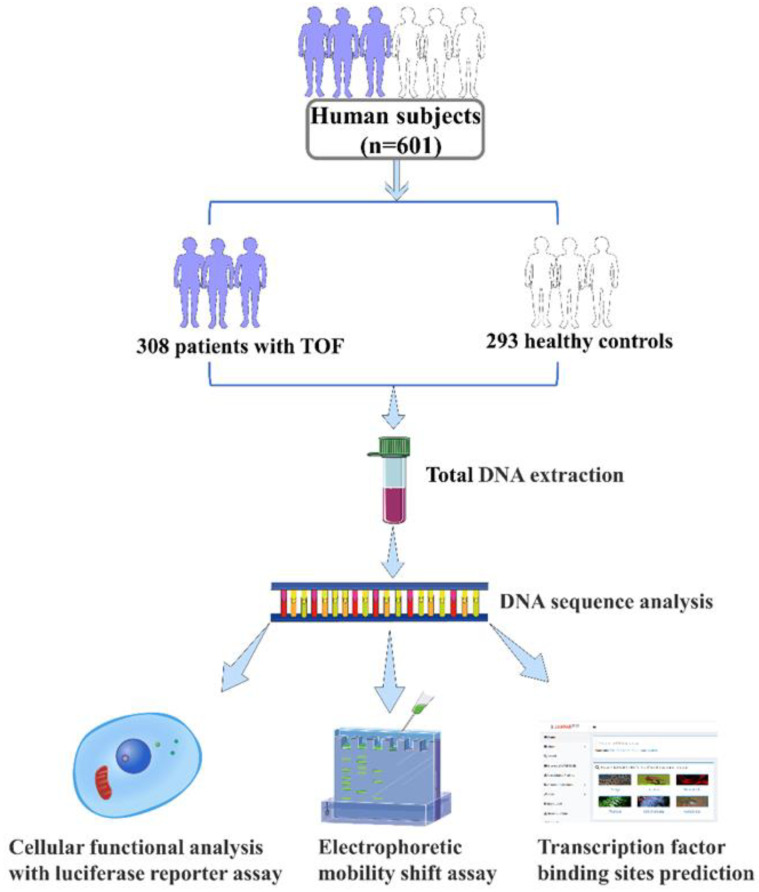
The flow chart of this study. A total of 601 subjects were enrolled in this study. Then we performed total DNA extraction, DNA sequence analysis, cellular functional analysis, electrophoretic mobility shift assay, and transcription factor binding sites prediction to test our hypothesis. TOF, Tetralogy of Fallot.

In this study, a total of 308 Chinese Han patients (175 males and 133 females; age range: 3 months to 19 years) diagnosed with TOF from the Department of Cardiovascular Surgery, TEDA International Cardiovascular Hospital, Tianjin University (Tianjin, China) and 293 Chinese Han healthy control subjects (165 males and 128 females; age range: 1 month to 14 years) with similar sex and age were recruited. We made an endeavor to recruit sporadic TOF patients without family history of CHD. All the normal control subjects were confirmed by echocardiography without CHDs or any other diseases. The investigation was in compliance with the outlined guidelines in the Declaration of Helsinki. The informed written consent was obtained from all subjects or their legal guardians prior to the study. Research protocol for this study was approved by the Ethics Committee of TEDA International Cardiovascular Hospital (Clinical Research Ethics Review Number: 2021-0715-4). The flow chart is shown in Figure 1.

### 2.2. ISL1 Promoter Sequence Analysis

Peripheral blood samples were collected according to the standard clinical procedure. Genomic DNA was extracted from peripheral blood for each TOF patient and control subject with TIANamp Blood DNA Kit (TIANGEN, China). The polymerase chain reaction (PCR) primers were designed based upon the reference sequence of the human *ISL1* gene in GenBank database (NCBI: NG_023040.1) as previously reported [14]. The promoter region of the human *ISL1* gene (1398 bp, from −1286 bp to +111 bp to the transcription start site) was amplified by PCR and directly bidirectionally sequenced. The primers for Sanger sequencing are listed in Table 1. Subsequently, the DNA sequences of above PCR products were compared with the wild-type *ISL1* promoter sequence.

### 2.3. Plasmid Constructs, Cell Culture, and Transfection

To functionally analyze the variants of *ISL1* gene promoter, DNA fragments of *ISL1* gene promoter with wild-type or variant-type, containing the terminal restriction sites KpnI and BglII, were generated by PCR and subcloned into the KpnI/BglII sites of the pGL3-basic to constitute expression vectors that were validated by Sanger sequencing. PCR primers are shown in Table 1.

Mouse cardiomyocyte (HL-1) cells were cultured in DMEM (Dullbecco’s modified Eagle’s medium, Gibco, Thermo Fisher, Waltham, MA, USA), to which was added 10% FBS (Fetal Bovine Serum, ExCell Bio, Shanghai, China), 100 μg/mL streptomycin (Beyotime, Shanghai, China), and 100 units/mL penicillin (Beyotime, Shanghai, China) in a 5% CO_2_ incubator at 37 °C. The above-constructed reporter plasmids with Lipofectamine™ 2000 Transfection Reagent (Invitrogen, Thermo Fisher, USA) were transfected into HL-1 cells. For standardizing the comparison between the variant-group and the wild-group, the expressing renilla luciferase reporter plasmid (pRL-SV40) was co-transfected into all groups as an internal control to normalize transfection efficiency. The empty pGL3-basic plasmid was utilized as a negative control.

### 2.4. Functional Analysis with Dual-Luciferase Reporter Assay

Transfected cells were harvested and lysed after 36 h. Firely and renilla luciferase activities of cells were measured with Dual Luciferase Reporter Gene Assay Kit (Beyotime, China) in the specified dual-luciferase reporter assay system (Thermo Scientific Fluoroskan FL, Thermo Fisher, Americia) according to the manufacturer’s protocol. The activities of *ISL1* gene promoter were assessed by the ratio of firefly luciferase activity to renilla luciferase activity. All experiments were performed in three independent replicates.

### 2.5. Nuclear Protein Extraction and Electrophoretic Mobility Shift Assay

To investigate the effect of variants within the promoter region of *ISL1* gene on the binding ability of transcription factors, electrophoretic mobility shift assay (EMSA) was performed with Chemiluminescent EMSA Kit (Beyotime, Shanghai China). Biotinylated double-stranded oligonucleotides based on the wild-type or variant-type sequence in *ISL1* gene promoter were utilized as probes. Biotin-labeled oligonucleotides for the EMSA experiment are shown in Table 1. Nuclear protein extracts from HL-1 cells and HEK-293 cells were prepared using the Nuclear and Cytoplasmic Protein Extraction Kit (Beyotime, Shanghai, China) and protein concentrations were determined with the Enhanced BCA Protein Assay Kit (Beyotime, Shanghai, China). The DNA-protein binding reaction with an equal amount of probe and nuclear extracts was performed at room temperature in 20 min. The mixture of DNA and protein mixed with sample loading buffer was then added to the sample wells of 4% polyacrylamide gel and transferred onto a nylon membrane (Beyotime, Shanghai, China). The oligonucleotides were cross-linked to the membrane using the UV light. Finally, biotinylated oligonucleotides were detected by chemiluminescence using the Chemiluminescent EMSA kit (Beyotime, Shanghai, China).

### 2.6. Transcription Factor Binding Sites Prediction

The online JASPAR database (http://jaspar.genereg.net, accessed on 7 August 2022) was used to analyze whether *ISL1* promoter region variants would alter the putative transcription factor binding site (TFBS). The relative profile score threshold was set at 85% in the JASPAR database.

### 2.7. Statistical Analysis

For continuous variables, results are expressed as mean ± SD. Non-parametric tests for independent samples were used to analyze the quantitative data and to calculate the statistical significance of the experimental results. The burden test for rare variants was performed by using SKAT package in R. The model parameters and residuals were calculated by ‘SKAT-Null-Model-MomentAdjust’ function for binary triat. ‘SKATBinary’ function was used for the burden test of a group of several rare variants [20]. The level of significance was set at *p* < 0.05. SPSS 19.0 software (IBM) was used for all statistical analyses.

## 3. Results

### 3.1. The Variants Identified in TOF Patients and Healthy Controls

**Figure 2:**
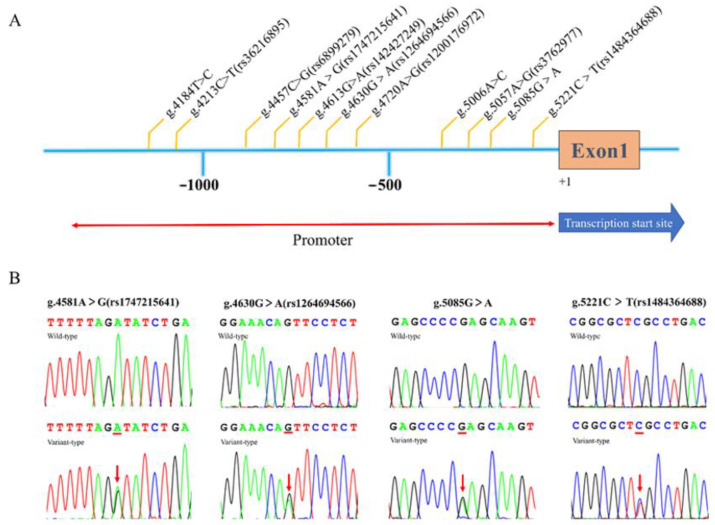
Identified variants of *ISL1* gene. (**A**) The locations of eleven variants identified within the *ISL1* gene promoter region in this study. The genetic variants were named according to the genomic DNA sequence of human *ISL1* gene (Genbank accession number NG_023040.1). (**B**) The sequencing chromatograms of the variants only found in patients with TOF. For all heterozygous variants [g.4581A > G(rs1747215641), g.4630G > A(rs1264694566), g.5085G > A, g.5221C > T(rs1484364688)], the top panel shows the wild−type and the bottom panel shows the variant-type. The variant site is labeled with the red line and arrow.

This study recruited 601 participants, including 308 TOF patients and 293 healthy controls (Figure 1). A total of eleven variants in *ISL1* gene promoter were identified by Sanger sequencing in these study subjects. The locations and details of these variants are presented in Figure 2A and Table 2. Among those eleven variants found in this study, one novel heterozygous variant [g.5085G > A] and three single-nucleotide variants (SNVs) [g.4581A > G(rs1747215641), g.4630G > A(rs1264694566), g.5221C > T(rs1484364688)] were found only in patients with TOF. The sequencing chromatograms of these four variants are presented in Figure 2B. In addition, the allele frequency of these four variants is less than 0.0001 in the NCBI ALFA database and GnomAD database and two of them [g.5085G > A, g.5221C > T(rs1484364688)] have 0 allele frequency (Table 2). Further, one of these four variants [g.4630G > A(rs1264694566)] was found in two patients. The above three SNVs and the novel variant [g.5085G > A] were further validated for cellular functional experiment.

There were four variants [g.4213C > T(rs36216895), g.4457C > G(rs6899279), g.4613G > A(rs142427249), g.5057A > G(rs3762977)] that were found in both TOF patients and control subjects (Table 2). Three variants [g.4184 T > C, g.4720 A > G (rs1200176972), g.5006 A > C] were only found in the healthy controls. These seven variants were excluded from the further study [21].

### 3.2. Functional Analysis of the Variants via Dual-Luciferase Reporter Assay

To further investigate the effect of variants on the transcriptional activity of the *ISL1* promoter, different reporter plasmids including a null pGL3-basic (negative control), wild-type *ISL1* gene promoter (pGL3-WT), and variant-type *ISL1* gene promoter (pGL3-4581G, pGL3-4630A, pGL3-5085A, pGL3-5221T) were constructed and transfected into the HL-1 cells, respectively. The dual-luciferase activities were assayed and relative activity of wild-type and variant *ISL1* gene promoter was examined.

**Figure 3:**
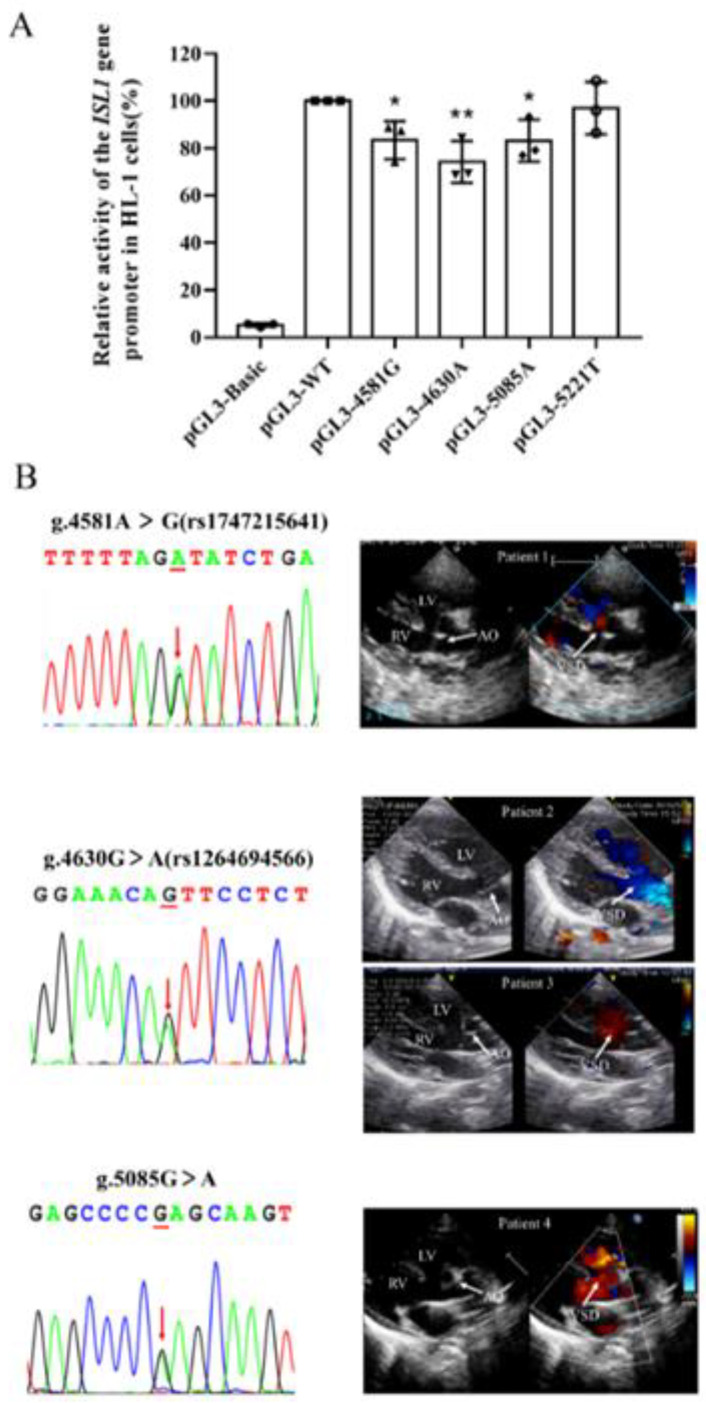
The result of the dual-luciferase reporter assay and the echocardiography of patients with these three variants. (**A**) Relative transcriptional activity of wild-type and variant-type *ISL1* gene promoter in HL-1 cells. The negative control was empty vector pGL3-basic and the transcriptional activity of the wild-type *ISL1* gene promoter was set as 100%. The quantitative data are presented as mean ± SD based on three independent experiments. * *p* < 0.05; ** *p* < 0.01. (**B**) The doppler echocardiography shows TOF in patients with variants of [g.4581A > G(rs1747215641), g.4630G > A(rs1264694566), g.5085G > A], showing the malalignment ventricular septal defect and aortic overriding. The ventricular septal defect is indicated with arrows. AO, aorta; RA, right atrium; RV, right ventricle; VSD, ventricular septal defect.

As illustrated in Figure 3A, dual-luciferase activity indicated that among the four expression vectors carrying genetic variants, three variants (4581G, 4630A, 5085A) significantly decreased the transcriptional activities compared with the wild-type (*p* < 0.05). In contrast, the rest one variant (5221T) did not significantly alter the transcriptional activity of the *ISL1* gene promoter (*p* > 0.05). The echocardiography of patients with variants is displayed in Figure 3B.

### 3.3. The Binding for Transcription Factors Affected by the Variants

**Figure 4:**
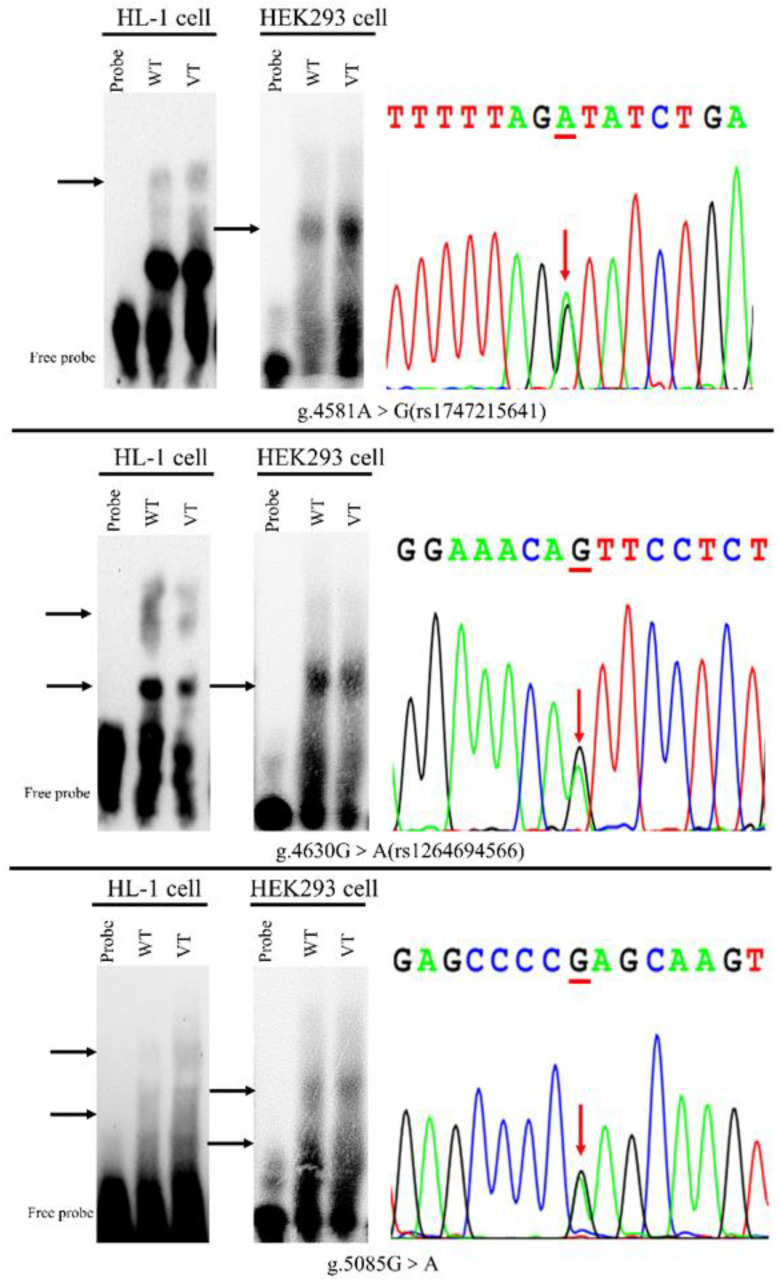
EMSA of biotin-labeled oligonucleotide-containing variants with nuclear extracts of HL-1 cells and HEK-293 cells. The amount of the labeled probe in VT and WT lane was the same. Free probe is at the bottom. The different binding for transcription factors between WT and VT is marked with arrows. Sequencing chromatograms of the three variants are represented at the right panel. WT, wild type, VT, variant type.

To experimentally investigate whether the binding ability of transcription factor was affected by the variants identified only in TOF patients, EMSA was performed with wild-type or variant biotinylated oligonucleotides. The above three variants (4581G, 4630A, 5085A) markedly affected the binding of transcription factor in both HL-1 and HEK-293 cells, as shown in Figure 4 (presented with arrows). In addition, similar to the result in dual-luciferase reporter assay, the variant (5221T) did not affect the binding of transcription factor (data not shown).

### 3.4. Variant-Affected Putative Binding Sites for Transcription Factor

**Figure 5:**
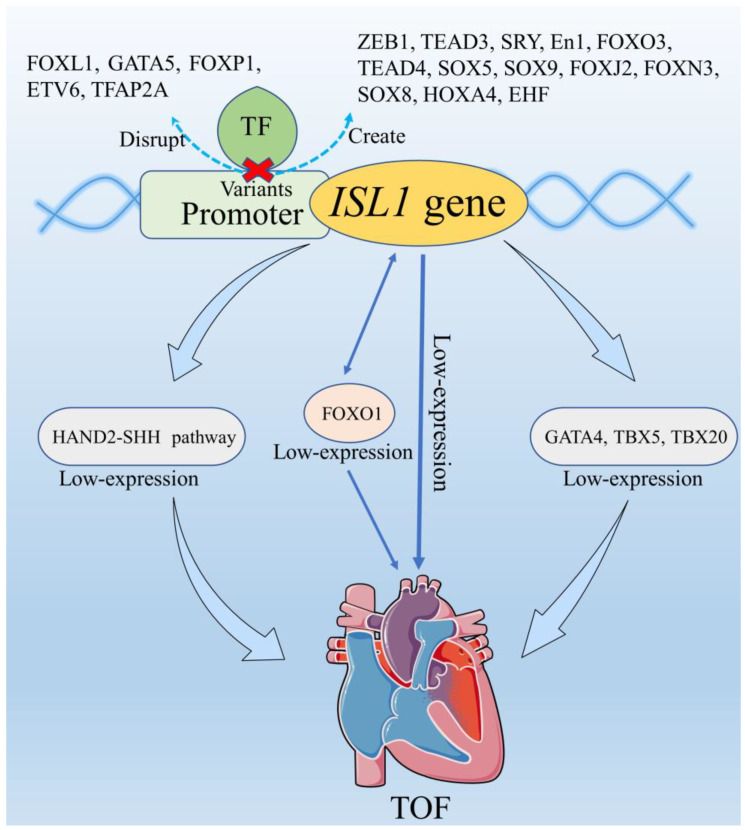
Schema describing the role of *ISL1* gene promoter region variants in the development of cardiac defect based on the results from the present study, the JASPAR database analysis, and the previous reports. Variants reduced *ISL1* gene promoter activity by disrupting and creating the TFBS (total of 32 [see Table 3]; 18 of them are involved in the heart development that are listed in the figure), contributing to the low expression of *ISL1*. Consequently, the low expression of *ISL1* may be directly involved in the formation of TOF and may reduce the expression of downstream target genes and pathway, which are related to the formation of TOF.

The online JASPAR database was fully utilized to further explore whether the binding sites for transcription factors were affected by variants identified in the *ISL1* gene promoter. As summarized in Table 3, the putative binding sites for transcription factor may be disrupted or created by variants identified in patients with TOF. The variant g.4581G may create new binding sites for *ZEB1*, *NKx3-1*, and *TEAD3*, and disrupt the binding sites for *FOXL1*, *RHOXF1*, and *GATA5*. Among these TFBSs, the mutations of *GATA5* have been reported in sporadic TOF patients. *SOX9*, a downstream target gene of *ISL1* gene, is regulated by *ISL1* gene through *ISL1-Fgf10-SOX9* pathway. In combination with the experimental results, the analysis by the JASPAR database, and literature reports, we established a schema (Figure 5) to illustrate the possible effect of the variants in *ISL1* gene promoter region and the roles of genes and pathways associated with *ISL1* gene in the etiology of TOF.

## 4. Discussion

The current study for the first time identified that (1) there are four variants within the *ISL1* gene promoter that are identified only in the TOF patients with 0 incidence in the control subjects and one of them (g.5085G > A) is novel; (2) three of these four variants (g.4581A > G, g.4630G > A, g.5085G > A) significantly altered the transcriptional activity of the *ISL1* gene promoter at cellular functional level; and (3) these three variants (g.4581A > G, g.4630G > A, g.5085G > A) causing functional changes evidently affected the binding of transcription factors verified by EMSA experiments. Therefore, these *ISL1* gene promoter variants (g.4581A > G, g.4630G > A, g.5085G > A) likely contribute to the development of TOF as risk factors.

The human *ISL1* gene, mapped to chromosome 5q11.1 and comprised with 349 amino acids, was first identified and cloned during the study of the regulation of insulin gene expression [22]. Recently, it has been reported that *ISL1* plays an important role in the regulation of the nervous system, the pancreas and cardiac development [23]. *ISL1* gene can be specifically expressed in cardiac progenitor cells. Decreased expression of the *ISL1* gene has been reported to be associated with many cardiovascular diseases in human, such as CHDs [24], atrial fibrillation [25], and dilated cardiomyopathy [26]. It has been reported that the copy number variations of *ISL1* gene is related to the etiology of TOF [27]. The expression of many genes is regulated through the binding of various transcription factors to promoter sequences [19]. To date, variants at the promoter region of human *ISL1* gene have not been illustrated in detail. Based on the above reports concerning *ISL1* gene, we hypothesized that variants in the *ISL1* gene promoter may affect the normal gene activation and lead to the disease. In this study, we analyzed the promoter of *ISL1* gene in patients with TOF and the variants were identified. The transcriptional activity analysis and EMSA results performed in the present study have clearly demonstrated that three variants discovered in *ISL1* gene promoter region notably changed the transcriptional activity, providing support for our hypothesis. The EMSA is an experiment that may demonstrate the interference (enhancement or suppression) of the binding affinity between transcription factors and promoter by variants. Different variants may affect the binding affinity for various transcription factors (see Table 3 for details) at the different levels, as shown in Figure 4.

It has been reported that variants in the gene promoter region create or disrupt TFBS at the promoter, affecting the transcriptional regulation of genes and putatively contributing to the development of disease. In the present study, three variants discovered in the *ISL1* gene promoter with altered cellular function were analyzed with bioinformatics through JASPAR database. We discovered that the variant g.4581A > G may disrupt the potential binding sites for *GATA5*, a member of the GATA family transcription factor that plays an essential role in the development of embryonic heart. Importantly, the *GATA5* loss-of-function mutation is associated with TOF [28]. In addition, the binding sites for SRY (sex determining region Y)-box9(*SOX9*) may be created by the variant g.4630G > A. The *ISL1–Fgf10–SOX9* network is a pivotal pathway in the embryonic lung branching morphogenesis and cell differentiation and *SOX9* is regulated by *ISL1* [29]. Further investigation to explore the relationship among *ISL1* gene promoter variants and these transcription factors is required.

Apart from *GATA5* and *SOX9*, among the 32 transcription factors listed in Table 3, at least 16 factors are closely related to the development of the heart. All those genes may be further studied with regard to the interaction to *ISL1* promoter in order to reveal the correlation among them and the pathological role in the development of TOF.

Taken together with the results from the present study, the JASPAR database analysis, and the previous reports, we established a schema (Figure 5) to summarize the possible role of *ISL1* gene promoter region variants in the development of TOF. Firstly, variants reduce *ISL1* gene promoter activity, contributing to the low expression of *ISL1*. Consequently, the low expression of *ISL1* may be involved in the formation of TOF. In addition, the activity of TOF-related genes, such as *GATA4* [30], *TBX5* [31], and *TBX20* [32], may be decreased by the low expression of *ISL1* [33]. Moreover, the low expression of *ISL1* may also lead to the reduced-expression of vascular endothelial growth factor related signaling gene *FOXO1*, which is interacted with *ISL1* and involved in the pathogenesis of TOF [34,35,36]. Finally, the low level of *ISL1* may reduce the expression of *HAND2* and *SHH* in the *HAND2-SHH* pathway, which is involved in the development of TOF [37,38,39].

There are some limitations in this study. The exact role of genotype–phenotype relationship needs to be further established. The precise role of the discovered *ISL1* promoter variants in the development of TOF requires further in vivo animal experiments. In addition, CHDs are developed with the interaction of related genes and the interaction between *ISL1* and other genes shown in Figure 5 needs to be further verified.

## 5. Conclusions

In conclusion, our study for the first time identified genetic variants in the promoter region of *ISL1* gene in patients with TOF. Furthermore, the variants were demonstrated to significantly alter the *ISL1* gene expression by the cellular functional experiment. Further EMSA experiment and bioinformatic analysis also demonstrated that these variants likely affect the binding of transcription factors and may contribute to the development of TOF as risk factors. Therefore, this study may provide new insights into the understanding of the promoter region and the genetic etiology involved in the development of CHD.

## Figures and Tables

**Table 1:** List of primers and oligonucleotides sequences for EMSA in this study.

Primers Name	DNA Sequences 5’-3’	Location	Position
PCR and sequencing primers			
*ISL1*-F1	5’-CTGTCTTTGGGAGACCGTAACA-3’	4039	−1286
*ISL1*-R1	5’-TGCCAATGCTGAAAGAGCCG-3’	5436	+111
Primers containing restriction sites			
*ISL1*-KpnI ^ab^	5’-(KpnI)-CGGGGTACCCTGTCTTTGGGAGACCGTAACA-3’	4039	−1286
*ISL1*-BglII ^ab^	5’-(BglII)-CAAGATCTTGCCAATGCTGAAAGAGCCGG-3’	5436	+111
The double-stranded biotinylated oligonucleotides for the EMSA			
Sequence variants	Oligonucleotides sequences		
g.4581 A > G	5’-CACCCAACGTTTTTAG(A/G)TATCTGAGGTTGGGG-3’		
g.4630 G > A	5’-TAGCAGTCAGGAAACA(G/A)TTCCTCTGGGTTTAATC-3’		
g.5085 G > A	5’-AACGGCCTGAGCCCC(G/A)AGCAAGTTGCCTCGGG-3’		
g.5221 C > T	5’-TGGGCGCGGCGCT(C/T)GCCTGACGTCCCCG-3’		

Note: The PCR primers are designed based on the genomic DNA sequence of the *ISL1* gene (NG_023040.1). The transcription start site is at the position of 5325 (+1). Abbreviations: F, forward; R, reverse; PCR, polymerase chain reaction; EMSA, electrophoretic mobility shift assay. ^a^ Restriction sites are underlined. ^b^ Protective bases are marked in bold. The underline indicates the restriction endonuclease site

**Table 2:** Variants within the *ISL1* gene promoter in TOF patients and controls.

Variants	TOF	Controls	Burden Test (*p*-Value)	Position ^a^	Genotypes	Allele Frequency		
Frequency in Control = 0 (Further Validation)						GnomAD	East Asian	
g.4581A > G(rs1747215641)	1	0	0.04	−744	AG	G = 0.00007	G = 0.00 *	G = 0.0000 ^#^
g.4630G > A(rs1264694566)	2	0		−695	GA	A = 0.000014	A = 0.00 *	A = 0.0003 ^#^
g.5085G > A	1	0		−240	GA	None	-	-
g.5221C > T(rs1484364688)	1	0		−104	CT	None	T = 0.00 *	-
Frequency in Control ≠ 0 (No Further Validation)						GnomAD		
g.4213C > T(rs36216895)	14	24	0.29	−1112	CT	None		
g.4457C > G(rs6899279)	14	24		−868	CG	G = 0.174		
g.4613G > A(rs142427249)	4	1		−712	GA	A = 0.00013		
g.5057A > G(rs3762977)	14	24		−268	AG	G = 0.149		

Abbreviations: -, not applicable; TOF, Tetralogy of Fallot. ^a^ Variants are located upstream (−) to the transcription start site of the *ISL1* gene at 5325 of NG_023040.1. * The Allele frequency of East Asian in ALFA database (Version: 20201027095038). ^#^ The Allele frequency of East Asian in GnomAD database (Accession: PRJNA398795).

**Table 3:** Predicted binding sites for transcription factors and promoter activity affected by variants.

Variants	Binding Sites for Transcription Factors		Promoter Activity
	Create	Disrupt	
g.4581A > G	ZEB1, NKx3-1, TEAD3	FOXL1, RHOXF1, GATA5	↓
g.4630G > A	SRY, En1, FOXO3, FOXG1, TEAD4, SOX5, SOX9, FOXD1, FOXJ2, FOXN3, SOX15, SOX8, HOXA4, GSX2, ETV2::DRGX	ELK4, FOXP1, ETV6, PRDM4	↓
g.5085G > A	EHF, TcfL5	Ahr::Arnt, TFAP2E	↓
g.5221C > T	-	TFAP2A, NFIX, HES1	No change

Abbreviations: -, not applicable.

## Data Availability

The individual SNP numbers are given in Table 2. The genetic variants described in this manuscript are available at https://www.ncbi.nlm.nih.gov/snp/ (accessed on 29 June 2022). Data supporting the findings of this study are available upon reasonable request from the corresponding authors.
